# New York Odontological Society

**Published:** 1885-12

**Authors:** 


### NEW YORK ODONTOLOGICAL SOCIETY.

Reported for the Independent Practitioner.

This Society held its regular monthly meeting on Tuesday evening, November 10th, in the parlors of the New York Academy of Medicine; President Jarvie in the chair.

After the regular order of business, Dr. Atkinson, at the request of the President, described an interesting case which recently came to his care, after having been under the treatment of physicians for a period of five years. The patient, a young girl, had suffered from a fistulous opening on the chin at the symphysis, and the almost constant discharge through this opening had not only impaired her health, but seriously interfered with her attendance at school. The long continuance of the trouble, and the fact that the young Miss was just verging into womanhood, caused her then attending physician to determine the extraction of the inferior incisors. To this resort the mother objected, and would not give her consent to such a sacrifice unless convinced that it was an absolute necessity. She sought the advice of Dr. Atkinson, taking her daughter with her. After a thorough examination, Dr. Atkinson delighted the mother by stating that, in his opinion, the fistula could be cured and the teeth saved, and he requested a consultation with both their attending physician and her former dentist, to take place at his office, the mother also to be present. The engagement was kept by all parties, and it was decided that openings should be made into the root canals of the incisor teeth. It was the desire of all present that Dr. Atkinson should perform the operation, and he drilled through the entire length of each tooth and root, and even through their foramina. Immediately after making the openings the characteristic indications of necrosed bone were made manifest. Dr. Atkinson described his treatment for disposing of the necrosed bone and clearing the fistulous tract, and stated that already a marked improvement had taken place, with every prospect of a permanent cure.

*Dr. F. Y. Clark*—Asked if medicines used in the treatment of such cases could not be forced through the root canal, without the necessity of drilling through the foramen.

*Dr. Atkinson*—Thought that in almost all such cases nests of microbes collected at the apex of the roots. These were broken up and destroyed only by drilling completely through the ends of such roots.

*Prof. C. N. Pierce,* of Philadelphia—Who was present, stated that in many cases it had been his practice to extract dead teeth, smoothly grind the apex of the roots, so often partly absorbed, insert into or fill the canals with wooden plugs saturated with a mixture of carbolic acid and tr. iodine, then replace the teeth and ligate the same to hold them in position until they become firm. This method, in the doctor's practice, had given good results. Dr. Pierce also stated that some time ago a lady patient called at his office who had just before received a fall in the street, completely knocking out her two superior central incisors, which were left where the mishap occurred. The doctor requested the lady to return immediately to the spot and secure, if possible, the lost organs. In a little time she came back, having been fortunate enough to find them. These were immediately placed in warm water; then the pulp canals were cleansed and filled with carbolized wood, and the teeth forced into their former positions. They again became firm and useful.

*Dr. S. G. Perry*—Was glad to hear Dr. Atkinson say that he drilled through the apex of roots, as he often did so, believing this was the only sure way to destroy the nests of microbes and to insure treatment that would reach the desired point.

*Dr. Clark*—Was greatly pleased to hear members of the Society speak of microbes, and organisms of decomposition; also their appreciation of antiseptic conditions. Not long since he remembered that, when speaking of these organisms, their existence was seemingly questioned, and his statements received with a smile.

*Dr. A. H. Brockway*—Presented a curious specimen of a supernumerary tooth in form of a bunch of nodules. Dr. Brockway then read a paper of an exceedingly practical nature on the "Rubber Dam," showing its utility, methods of adjustment, ligatures, clamps, and weights for holding the rubber in position. He preferred a dark rather than a light color of rubber, as it caused the teeth to stand out in stronger contrast against a dark background.

*Dr. Perry*—Found a piece of rubber dam rolled in a napkin very useful in preventing moisture working through, when used in operations for the upper teeth.

*Dr. W. T. Laroche*—In order to prevent the possibility of moisture oozing up between the teeth, often uses two thicknesses of the rubber, punching each and putting one piece over the other.

*Prof. Pierce*—Read a somewhat lengthy paper entitled "Function; its Evolution and Influence on Organization."

The essay was listened to with marked attention and much interest, and for which the thanks of the Society were gratefully tendered. The paper was briefly discussed by Drs. Atkinson, J. Morgan Howe and J. Smith Dodge, Jr.

